# Supplementary material for: Copper and Zinc Sulfates Suppress Streptomyces spp. and Enhance Potato Resistance via Thaxtomin A Inhibition and Defense Gene Regulation
Source: Microorganisms. 2025 May 31;13(6):1288. doi: 10.3390/microorganisms13061288 (PMC12195330; doi:10.3390/microorganisms13061288)
Supplement: Supplementary file 1 [file microorganisms-13-01288-s001.zip › microorganisms-3588867-supplementary.pdf]

Table S1. The inhibitory effects of copper sulfate and zinc sulfate solution on five kinds of *Streptomyces* spp. under different concentrations.

Treatme n-ts	Concentra ti-on (g/mL)	Diameter of inhibition zone (cm)				
		**HRB-1** *S. scabies*	**HL-12** *S. rochei*	**JX-15** *S. lavendulae*	**JMS-3** *S. acidiscabies*	**AC-15** *S. bottropensis*
$CuSO_4$	0.00	0.00±0.00 e	0.00±0.00 f	0.00±0.00 e	0.00±0.00 f	0.00±0.00 d
	0.04	2.45±0.19 d	0.91±0.04 e	2.18±0.08 d	1.13±0.03 e	2.45±0.14 c
	0.07	2.88±0.11 c	1.16±0.08 d	2.90±0.61 c	1.93±0.05 d	2.69±0.04 c
	0.10	3.13±0.26 bc	1.61±0.20 c	3.54±0.13 b	2.63±0.07 c	3.04±0.05 b
	0.13	3.36±0.14 b	2.18±0.06 b	4.41±0.15 a	3.30±0.04 b	3.50±0.19 a
	0.16	3.79±0.21 a	2.74±0.07 a	4.62±0.09 a	3.88±0.06 a	3.76±0.24 a
$ZnSO_4$	0.00	0.00±0.00 d	0.00±0.00 f	0.00±0.00 f	0.00±0.00 e	0.00±0.00 e
	0.10	3.00±0.34 c	1.57±0.09 e	2.25±0.06 e	1.60±0.14 d	1.79±0.48 d
	0.20	3.42±0.11 b	2.29±0.05 d	2.68±0.10 d	1.95±0.07 c	2.27±0.04 c
	0.30	3.87±0.05 a	2.81±0.07 c	3.15±0.24 c	2.09±0.08 c	2.69±0.13 b
	0.40	4.11±0.20 a	3.33±0.02 b	3.55±0.26 b	2.69±0.21 b	3.11±0.02 a
	0.50	4.20±0.22 a	3.60±0.07 a	3.92±0.19 a	3.68±0.15 a	3.37±0.09 a

Note: Small letters (a, b, c, etc.) in each column indicate significant differences ($p < 0.05$) among different concentrations of $CuSO_4$ or $ZnSO_4$ for the same Streptomyces strain, as determined by one-way ANOVA followed by Tukey's post-hoc test. The same letters within a column indicate no significant difference ($p \geq 0.05$).

Table S2. Toxin content produced by Streptomyces scabies under treatments with different medicament concentrations.

Treatments	Thaxtomin A toxin levels (mg/L)
CK	487.75±0.34 a
0.001 g/mL $CuSO_4$	359.73±0.83 d
0.0015 g/mL $CuSO_4$	267.53±0.43 f
0.002 g/mL $CuSO_4$	209.65±0.04 g
0.002 g/mL $ZnSO_4$	423.60±0.21 b
0.0035 g/mL $ZnSO_4$	364.93±0.18 c
0.005 g/mL $ZnSO_4$	286.36±0.26 e

Table S3. Growth effect of copper sulfate and zinc sulfate on potato plants.

Treatments	Concentration (g/mL)	Plant height (cm)	Stem diameter (cm)	Length of underground (cm)	Lateral root length (cm)	Lateral root number (s)	Stolon number (s)	Fresh weight (g)	Dry weight (g)
CK	0.000	40.24±1.36 bc	0.98±0.07 cd	3.60±0.05 d	16.30±3.36 c	31.67±6.72 a	7.43±1.13 b	98.95±10.06 c	29.91±2.36 d
	0.001	39.23±2.36 c	0.95±0.08 d	4.43±0.15 b	19.47±2.38 abc	31.00±4.17 a	7.86±0.69 ab	99.38±9.98 c	29.61±3.19 d
CuSO4	0.0015	41.39±3.34 abc	1.09±0.07 ab	3.67±0.08 d	20.33±2.22 ab	30.00±5.91 a	8.43±1.13 ab	107.66±15.39 bc	34.86±5.61 bc
	0.002	42.56±1.49 ab	1.13±0.03 a	4.21±0.21 c	21.03±1.33 a	30.33±4.46 a	9.14±0.69 a	119.41±16.96 ab	37.33±4.66 b
	0.002	40.67±2.22 abc	1.03±0.04 bc	4.17±0.14 c	17.27±2.31 bc	30.67±7.17 a	7.86±1.68 ab	111.31±18.33 abc	31.07±2.71 cd
ZnSO4	0.0035	41.56±3.31 abc	1.08±0.04 ab	5.57±0.14 a	19.50±1.74 abc	30.33±3.25 a	8.43±1.81 ab	118.72±23.11 ab	36.85±3.33 b
	0.005	43.48±3.22 a	1.10±0.05 a	4.01±0.13 c	20.73±5.33 ab	30.67±3.51 a	9.05±0.82 a	126.14±9.74 a	42.25±3.94 a
0.00035g/mL Streptomycin	0.00035	40.07±2.11 bc	1.03±0.05 bc	3.73±0.33 d	20.52±3.19 ab	30.81±4.84 a	7.83±1.41 ab	101.97±10.39 bc	31.17±4.33 cd

Note: Values represent mean ± standard error (SE) from three biological replicates. Different lowercase letters (a, b, c, etc.) within each column indicate significant differences among treatments ($p < 0.05$), as determined by one-way ANOVA followed by Tukey's post-hoc test.

Table S4. Copper and Zinc content in the soil.

Treatments	Concentration (g/mL)	Soil content (mg/kg)
CK	0.00	23.04±0.43 d
	0.001	56.90±1.44 c
$CuSO_4$	0.0015	66.26±0.98 b
	0.002	74.09±1.13 a
CK	0.00	15.44±1.00 d
	0.002	34.72±1.02 c
$ZnSO_4$	0.0035	66.55±0.95 b
	0.005	94.67±0.95 a

Table S5. Primer sequence.

Primers	Nucleotide sequence (5，-3，)	base number (bp)
Actin-F	AGCATCCTGTCCTCCTAACTGA	22
Actin-R	ACACCATCACCAGAGTCCAAC	21
PR1-F	GGGAGAAGCCAAACTACAACTA	22
PR1-R	TTGCATGAAATGAACCACCA	20
PR3-F	TCAGAAAGATACTATGGCAGAGGAC	25
PR3-R	TGTGGCTACTAAATCAGGGTTGTT	24
PR9-F	CTGAAACCCTTGCAGTTATGAT	22
PR9-R	ATCTTGCTCTTCCAAATGTATGT	23
SOD1-F	AAACAGGTCGAAAAGCCCGT	20
SOD1-R	ACTTGAGCCAAGCGAACAACAC	22
HSF1-F	GCAAAATCAAATGTTGGTGT	20
HSF1-R	AAATGGGTCTTCTTGTGGG	19

Annex

Gene & Accession Numbers:

Genes studied:

PR1 (Pathogenesis-related protein 1)
PR3 (Chitinase)
PR9 (Peroxidase)
SOD1 (Superoxide dismutase 1)
HSF1 (Heat shock factor 1)

Abbreviations:

CAT: Catalase
$CuSO_4$: Copper sulfate
HPLC: High-performance liquid chromatography
MDA: Malondialdehyde
POD: Peroxidase
PPO: Polyphenol oxidase
qPCR: Quantitative real-time PCR
SOD: Superoxide dismutase
$ZnSO_4$: Zinc sulfate
